# The Association of Fit-Fat Index with Incident Diabetes in Japanese Men: A Prospective Cohort Study

**DOI:** 10.1038/s41598-017-18898-3

**Published:** 2018-01-12

**Authors:** Robert A. Sloan, Susumu S. Sawada, Lee I-Min, Yuko Gando, Ryoko Kawakami, Takashi Okamoto, Koji Tsukamoto, Motohiko Miyachi

**Affiliations:** 1 Kagoshima University, Graduate Medical and Dental School, Department of Psychosomatic Internal Medicine, Kagoshima, Japan; 2 National Institutes of Biomedical Innovation, Health and Nutrition, Department of Health Promotion and Exercise, Tokyo, Japan; 3 Harvard Medical School, Brigham and Women’s Hospital, Boston, MA USA; 4 Harvard T.H. Chan School of Public Health, Department of Epidemiology, Boston, MA USA; 5 Waseda University, Faculty of Sport Sciences, Saitama, Japan; 6 Tokyo Gas Co., Ltd., Health Promotion Center, Tokyo, Japan

## Abstract

Type 2 diabetes is increasing globally and in Asia. The purpose of this study was to examine the association of a fit-fat index (FFI) with diabetes incidence among Japanese men. In total 5,014 men aged 18–64 years old, who had an annual health check up with no history of major chronic disease at baseline from 2002 to 2009 were observed. CRF was estimated via cycle ergometry. Overall, 7.6% of the men developed diabetes. The mean follow-up period was 5.3 years. Hazard ratios, 95% confidence intervals and P trend for diabetes incidence were obtained using the Cox proportional hazards model while adjusting for confounding variables. High FFI demonstrated lower risk 0.54 (0.36–0.82) compared to low BMI 0.63 (0.44–0.90), low WHtR 0.64 (0.41–1.02), and High CRF 0.72 (0.51–1.03). FFI showed a marginally stronger dose response relationship across quartiles (P (trend) =0.001) compared to BMI (P (trend) =0.002), WHtR (P (trend) =0.055), and CRF (P (trend) =0.005). Overall, both fitness and fatness play independent roles in determining diabetes incidence in Japanese men. FFI may be a more advantageous physical fitness measure because it can account for changes in fitness and/or fatness.

## Introduction

According to officials at the International Diabetes Federation, the prevalence of diagnosed and undiagnosed Type 2 diabetes (*herein diabetes*) in Japanese adults is 11%^1^. Concomitantly obesity and physical inactivity prevalence are reported to be 3.5% and 38%, respectively^2^. To slow the rise, the Japanese Ministry of Health, Labor and Welfare is making efforts aimed at reducing metabolic syndrome and increasing participation rates for medical screening. Though beneficial, such interventions tend to use more traditional health care approaches rather than health promotion strategies designed to identify and act upon earlier stage risk. Phenotypically, Asian populations have greater predisposition towards diabetes, because they tend to store more visceral adiposity and have less muscle mass for a given body mass index (BMI)^3^. To complement current efforts, practical upstream approaches targeting components of health-related physical fitness are also needed to help identify and lower diabetes risk.

Cardiorespiratory fitness (CRF) and waist-to-height-ratio (WHtR) are objective components of fitness and fatness linked to diabetes risk^4,5^. CRF is an indicator of habitual moderate-to-high intensity aerobic physical activity and depends on the combined function of respiratory, cardiovascular, and musculoskeletal systems^6,7^. Moreover, prediabetic individuals with higher CRF levels have lower mortality risks, regardless of fatness levels^8^. Unlike other anthropometric measures, WHtR uses a standard boundary value (<0.50) across phenotype, ethnicity, gender, and age, allowing for better comparability between populations^5^. Although BMI and WHtR are correlated, they are different. WHtR is a surrogate measure of visceral adiposity, and BMI is a surrogate measure of total lean and fat mass. Investigators have also proposed WHtR as a better screening tool than waist circumference and BMI for adult cardiometabolic risks^9^.

Epidemiological and experimental studies have indicated fitness and fatness have independent roles in delaying or preventing the onset of diabetes^10–12^. Findings from systematic reviews of clinical trials have shown diet and/or exercise impact fatness, which in turn may affect diabetes incidence. Conversely, diet and/or exercise may affect diabetes incidence independent of fatness. Notably, the use of self-reported diet and exercise is a limitation across trials^13,14^. Researchers investigating biological mechanisms suggested fitness and fatness can impact the onset of diabetes across an array of mechanisms by varying degrees, depending on intensity, type, and duration of the intervention^7,15–17^. Therefore, researchers have called for more investigations to focus on the *combined* influences of fitness and fatness^11,18,19^.

Considering the evidence for varying levels of fitness and fatness to impact diabetes incidence^10–12^, researchers have begun investigating the utilization of a comprehensive fit-fat index (FFI)^20–24^ on health outcomes. FFI quantifies a given degree of CRF for a given degree of WHtR (CRF ÷ WHtR). Less fat individuals are not always more fit and conversely fitter individuals are not always less fat. A less fit/less fat individual may have the same risk as a more fit/more fat individual^12^.

To date, no researchers have investigated the independent or combined relationship of objectively measured CRF and WHtR with diabetes incidence in an Asian population. We examine the association of FFI as it relates to diabetes incidence in a prospective cohort study of Japanese men. We hypothesized high FFI would associate with lower diabetes incidence better than high fitness or low fatness.

## Results

The average follow-up period was 5.3 years (ranging 1 to 7 years); 7.6% of the men developed incident diabetes, and 17.5% of the men reported a family history of diabetes. Compared with nondiabetic men, the men who developed diabetes tended to be older, with lower fitness, higher fatness, higher systolic blood pressure, higher prevalence of family history, and were smokers (Table 1). Overlap of the quartiles is due to FFI being correlated with fit fat variables (Supplementary Table 1). Independent associations between fitness and fatness covariates, and diabetes incidence are shown in Table 2; total man-years and cases were included. High FFI demonstrated lower risk compared to high CRF, low BMI and low WHtR. Compared to CRF, WHtR and BMI, the magnitude and direction of association was marginally stronger between FFI and diabetes incidence. Diabetes incidence was also independently associated with age, systolic blood pressure, smoking behavior, and family history (Supplementary Table 2).

**Table 1:** Baseline Characteristics of Participants.

Characteristics	All participants	Diabetes	Nondiabetic	P value
n	5,014	351	4,663	—
Age (years)	48.5 ± 8.1	49.9 ± 5.8	48.4 ± 8.2	<0.001
FFI	79.4 ± 20.0	70.8 ± 16.7	80.0 ± 20.0	<0.001
CRF (ml/kg/min)	38.6 ± 7.6	36.0 ± 6.6	38.8 ± 7.6	<0.001
WHtR	0.49 ± 0.04	0.52 ± 0.05	0.49 ± 0.04	<0.001
BMI (kg/m^2^)	23.7 ± 2.9	25.0 ± 3.4	23.6 ± 2.8	<0.001
Systolic blood pressure (mmHg)	125.4 ± 14.4	130.7 ± 15.2	125.0 ± 14.2	<0.001
Current smokers, *n* (%)	2,410 (48.0)	196 (55.8)	2,214 (47.5)	0.003
Current drinkers, *n* (%)	4,309 (85.9)	298 (84.9)	4,011 (86.0)	0.577
Family history of diabetes, *n* (%)	879 (17.5)	88 (25.1)	791 (17.0)	<0.001

FFI: Fit-Fat Index (METs ÷ WHtR), CRF: Cardiorespiratory Fitness, WHtR: Waist-to-Height Ratio (Waist cm ÷ Height cm), BMI: Body Mass Index. Data are means ± SD, unless otherwise specified.

**Table 2:** Adjusted hazard ratios of diabetes incidence by fitness and fatness factors.

Potential risk factors	n	Man-years	Cases	Age-adjusted	Multivariable-adjusted	P value
				HR (95%CIs)	HR (95%CIs)	
FFI^a^						
1st quartile (Low)	1,254	5,919	149	1.00 (Referent)	1.00 (Referent)	—
2nd quartile	1,251	6,572	91	0.60 (0.46–0.78)	0.77 (0.59–1.02)	0.068
3rd quartile	1,257	6,836	70	0.46 (0.35–0.62)	0.67 (0.49–0.92)	0.013
4th quartile (High)	1,252	7,321	41	0.29 (0.21–0.42)	0.54 (0.36–0.82)	0.003
				P for trend <0.001	P for trend = 0.001	
CRF^b^						
1st quartile (Low)	1,301	6,113	149	1.00 (Referent)	1.00 (Referent)	—
2nd quartile	1,255	6,729	84	0.57 (0.44–0.75)	0.67 (0.51–0.88)	0.005
3rd quartile	1,376	7,532	68	0.44 (0.33–0.58)	0.57 (0.42–0.77)	<0.001
4th quartile (High)	1,082	6,274	50	0.44 (0.32–0.61)	0.72 (0.51–1.03)	0.073
				P for trend <0.001	P for trend = 0.005	
WHtR^c^						
1st quartile (High)	1,248	6,170	152	1.00 (Referent)	1.00 (Referent)	—
2nd quartile	1,218	6,312	83	0.55 (0.42–0.72)	0.76 (0.56–1.03)	0.072
3rd quartile	1,258	6,807	72	0.47 (0.35–0.62)	0.75 (0.53–1.06)	0.098
4th quartile (Low)	1,290	7,359	44	0.30 (0.21–0.42)	0.65 (0.41–1.02)	0.059
				P for trend <0.001	P for trend = 0.055	
BMI^d^						
1st quartile (High)	1,252	6,551	153	1.00 (Referent)	1.00 (Referent)	—
2nd quartile	1,255	6,640	72	0.43 (0.33–0.57)	0.52 (0.39–0.70)	<0.001
3rd quartile	1,254	6,681	65	0.40 (0.30–0.54)	0.55 (0.40–0.75)	<0.001
4th quartile (Low)	1,253	6,776	61	0.38 (0.28–0.51)	0.63 (0.44–0.90)	0.011
				P for trend <0.001	P for trend = 0.002	

All potential risk factors were adjusted for age (continuous variable), systolic blood pressure (continuous variable), drinking habit (none, 1–2 times/week, 3–4 times/week, and ≥5 times/week), smoking habit (nonsmokers, past smokers, 1–10 cigarettes/day, 11–20 cigarettes/day, and ≥21 cigarettes/day), and family history of diabetes (present or not). ^a^adjusted for body mass index (continuous variable). ^b^adjusted for waist-to-height ratio (continuous variable), and body mass index (continuous variable). ^c^adjusted for body mass index (continuous variable), and cardiorespiratory fitness (continuous variable). ^d^adjusted for fit-fat index (continuous variable).

## Discussion

The current prospective cohort study was used to investigate the associations between fitness and fatness with diabetes incidence in a large cohort of healthy Japanese men from baseline. Consistent with the literature, our findings indicated fitness and fatness are useful in showing the probability of diabetes incidence, but combined fitness and fatness offered a slightly more refined risk marker^12,25^. To the best of our knowledge, this is the first study to evaluate the associations between objectively measured combined and independent CRF and WHtR with diabetes incidence in an Asian population.

Findings from a large 25-year prospective cohort study of Caucasian men indicated discriminative clinical values across fitness and fatness measures to be similar. Regarding diabetes incidence, FFI exhibited lower (higher) incident risks in cases in which independent measures of fitness or fatness showed a higher (lower) risk. The investigators also implied individuals with similar FFI scores and incident risk may be classified in different joint categories^12^. Loprinzi and Edwards conducted an investigation using a representative American sample from the National Health and Nutrition examination survey. Loprinzi and Edwards found lower FFI to independently associate with inflammatory markers and for every 1 unit increase in FFI, a corresponding significant decrease in C-reactive protein (β = −0.016; −0.02, −0.006) occurred^21^.

Few epidemiological studies have provided evidence for the joint associations of objectively measured fitness and fatness with diabetes incidence. By category, unfit/fat has the highest risk, unfit/lean and fit/fat groups have lower risk and fit/lean has the lowest risk^11^. Similar findings were recently found in a prospective study with Korean men whereby joint relationships between CRF and BMI demonstrated unfit/fat men had nearly a 2-fold increased risk for diabetes when compared to fit/lean men^26^. There was no increased risk among fit/fat and unfit/lean men. Conversely, in a group of Japanese men, a joint analysis indicated BMI played a substantially larger role than CRF when considering the likelihood of diabetes^27^. However, misclassification bias for fitness and fatness across joint categories may have occurred.

Notably, in our study we found men within the second quartile with higher BMI to have lower incidence of diabetes than those in the fourth quartile (Low BMI). This was likely due to the effects of confounding factors. Furthermore, in a separate study, Hsieh and colleagues found 46% of normal BMI Japanese men to be at higher risk because they had elevated WHtR levels^28^. These types of inconsistencies may provide argument for the use of a more comprehensive measure such as FFI.

Though fitness and fatness are commonly linked to diabetes, the causal biological mechanisms are not entirely understood. Chronic adaptions to physical activity may improve CRF and/or visceral adiposity without changes in BMI and changes in energy balance may reduce visceral adiposity without a change in BMI or CRF^16,17^. Biochemical and structural adaptations to habitual physical activity may occur in obese and nonobese individuals, resulting in adaptations in peripheral skeletal muscle glucose metabolism, hepatic function, adipose tissue, insulin action, inflammation, and HbA1_c_^7,15,29^. Reduction of visceral fatness may reduce HbA1c, insulin resistance, inflammation, and excess free fatty acids^16,17^.

The strengths of our study included the use of valid and objective measures at baseline and follow-up, such as blood glucose, CRF, waist girth, height, and weight. Although CRF was estimated, previous studies have shown a high correlation between laboratory measured CRF and the submaximal Åstrand Ryhming protocol^30^. The generalizability of the findings was limited, because participants were Japanese men, ages 18 to 64 years old, working at the same company. Conversely, the homogeneity of the sample strengthened the internal validity of our findings by limiting demographic confounders. In future studies, we plan to test the relevancy of FFI for diabetes risks in women. CRF, WHtR and BMI data were obtained during baseline examination; therefore, we cannot account for changes during the follow-up period that were not considered. We were also not able to account for any related medical therapy that may have impacted diabetes incidence. Lastly, the average follow-up of 5.3 years may have been too short to detect the potential impact of fitness and fatness over time.

## Methods

### Participants

The research ethics committee of the National Institutes of Biomedical Innovation, Health and Nutrition (NIBIOHN) approved the current study methodology, protocol, and procedures. The health examinations were done under Japanese Industrial Safety and Health Law. All information from the health examinations was used only in aggregate form without reference to or disclosure of individual information. For the purpose of the current study, a de-identified limited data set was collected used and approved by the NIBIOHN. A detailed description of the methods and protocols used in this investigation is already published, new additions or variations have been stated^31^.

The participants were employees of a company that supplies natural gas to the greater Tokyo area. The Japan Industrial Safety and Health Law officials require all employees undergo annual health examinations. The participants were 6,884 workers who underwent a health examination and graded exercise test between April 2002 and March 2003. Females were excluded from the current study because of the small number of female participants (*n* = 831). Also, 540 men with diabetes were excluded, along with 499 males based on a lack of data regarding potential confounders. As participants in the current study, a total of 5,014 men, ages 18 to 64 years old, were followed until March 2009. The company to which the participants belonged has adopted the mandatory retirement system, which has been introduced by many companies in Japan. In this system, the employment relationship is automatically completed when the worker’s age reaches a specific age (this company: 60 years). However, after retirement, there is another system to employ workers as part-time employees until 65 years of age based on their wishes. Most workers continue to work until 65 years of age. On the other hand, some workers retire at the age of 50 years or older, utilizing systems to promote early retirement, such as a system for job-change to affiliated companies and redundancy pay system. Thus, the study participants were to retire between 50 and 65 years of age (n = 1,902) during the follow-up period. At the point of retirement, follow-up was discontinued (censored case).

### Health Examination

The annual health examinations, including objective measurements of height, body weight, and blood pressure occurred between April 2002 and March 2003. An automated sphygmomanometer measured the resting blood pressure with the participant in a sitting position. Furthermore, we investigated the potential confounders regarding diabetes development of the participants, including cigarette smoking, alcohol intake, and family history of diabetes, using a self-administered questionnaire.

### Determination of Diabetes Incidence

Diabetes incidence was determined based on health examinations conducted annually from April 2002 to March 2009. The criteria for the diagnosis of diabetes were based on the diagnostic guidelines of the American Diabetes Association and the Japan Diabetes Society^32,33^. Participants with fasting blood glucose levels exceeding 7.0 mmol/L (126 mg/dL) were regarded as having diabetes. The fasting condition was confirmed by a verbal confirmation. From 2007, hemoglobin A1c (HbA1c; NGSP) levels were also used to determine the year in which diabetes was developed. As with fasting blood glucose levels, when employees exhibited HbA1c levels of ≥6.5% (48 mmol/mol), the year in which they had developed diabetes. Furthermore, using a questionnaire administered during examinations, we asked whether a physician had made a diagnosis of diabetes. If more than one of the criteria were met, the year of the earliest episode was indicated as the year of diabetes development.

### Cardiorespiratory fitness

The estimated maximal oxygen uptake (ml/kg/min), which is an index of CRF, was measured with a submaximal exercise test on a cycle ergometer (Monark Exercise AB, Vansbro, Sweden). The exercise test was composed of a maximum of three steps, each lasting 4 min, with increased resistance in each step. The heart rate was measured based on the R-R interval on the electrocardiogram. The target heart rate was established as 85% of age-predicted maximum heart rate (220 minus age in years). The load was increased 37 watts per step until the target heart rate was reached. CRF was estimated using the Åstrand-Ryhming nomogram^34^ and Åstrand age-correction factors^35^. The method of estimating CRF used in the current study has been shown to strongly correlate with results determined using a direct method, according to comparative studies^30,36^. A detailed description of the protocol has been already published pertaining to this cohort^31^.

### Anthropometric measures

The height and body weight of all participants were measured at health examinations conducted between April 2002 and March 2003. A scale was used to measure the body weight with the participants in light clothing and with shoes removed. The body mass index (BMI) was calculated as weight in kilograms divided by the square of the height in meters (kg/m^2^). Waist circumference was measured at the top of the right iliac crest with a nonelastic tape measure to the nearest 0.1 cm. WHtR was calculated by dividing waist in cm by height in cm.

### Fit-Fat index

Fit-fat index (FFI) represents the combination of CRF as the estimated VO^2^max and WHtR (VO^2^max ÷WHtR) expressed in the form of a quotient. Higher scores are considered better and generally range from ~30 to 150 on a continuous scale. FFI compares individuals beyond independent or joint categories for CRF and WHtR (i.e., Unfit/lean, 32 ml/kg/min ÷ 0.45 WHtR = 70 FFI; Fit/ Fat 38.5 ml/kg/min ÷ 0.55 WHtR = 70 FFI). Depending on the type of CRF test, FFI can also be determined by dividing maximum metabolic equivalent (MET) by WHtR^12^.

### Statistical Analysis

We compared baseline characteristics of participants with and without diabetes incidence. We showed the mean and standard deviation for continuous variables and the percentage for category variables. Also, we compared both groups using a *t*-test for continuous variable and Fisher’s exact test for categorical variables, as appropriate. Cox proportional hazards models were used to estimate independent effects across quartiles of fitness and fatness on diabetes incidence. Fitness fatness variables were correlated (Supplementary Table 1), therefore all models were designed to mitigate overadjustment bias and multicollinearity^37^. Each model was adjusted for age, systolic blood pressure, cigarette smoking, alcohol intake, and family history of diabetes. CRF and WHtR models were adjusted for respectively and included BMI. FFI and BMI models were adjusted for respectively and did not include CRF. The lowest quartile was used as the reference group for FFI and CRF models, and the highest quartile was used as the reference group for WHtR and BMI models. In separate analyses, Cox proportional hazards models were used to estimate independent effects across confounders (Supplementary Table 2).

The proportionality assumption of the models was tested using a log-minus-log plot; no evidence of violation was found. The Statistical Package for Social Science (SPSS) software was used for statistical analysis (SPSS, Inc., Chicago, Illinois, USA). In the analysis, *p* values < 0.05 were two-sided and were considered statistically significant.

### Data Availability

The data that support the findings of this study are available from Tokyo Gas Co, Ltd. but restrictions apply to the availability of these data, which were used under license for the current study, and so are not publicly available. Data are however available from the authors upon reasonable request and with permission of Tokyo Gas Co, Ltd.

## Conclusions

In summary, our findings add to and expand upon the dearth of evidence regarding the combined relationship of objectively measured fitness and fatness with diabetes risk. Using practical methods of physical fitness assessment, we found FFI to be a more useful etiological marker of diabetes incidence in a large cohort of Japanese men. In future studies, researchers should examine FFI utility among women, other health outcomes, and in experimental investigations.

## Electronic supplementary material

Supplementary Tables
