# Supplementary material for: The Association of Fit-Fat Index with Incident Diabetes in Japanese Men: A Prospective Cohort Study
Source: Sci Rep. 2018 Jan 12;8:569. doi: 10.1038/s41598-017-18898-3 (PMC5766556; doi:10.1038/s41598-017-18898-3)
Supplement: Supplementary file 1 — Supplementary Tables [file 41598_2017_18898_MOESM1_ESM.docx]

**Supplementary Information**

**The Association of Fit-Fat Index with Incident Diabetes in Japanese Men: A Prospective Cohort Study**

Robert A. Sloan^1^, Susumu S. Sawada^2^, Lee I-Min^3, 4^, Yuko Gando^2^, Ryoko Kawakami^5^, Takashi Okamoto^6,^ Koji Tsukamoto^6^, Motohiko Miyachi^2^

^1^ Kagoshima University, Graduate Medical and Dental School, Department of Psychosomatic Internal Medicine, Kagoshima, Japan

^2^ National Institutes of Biomedical Innovation, Health and Nutrition, Department of Health Promotion and Exercise, Tokyo, Japan

^3^ Harvard Medical School, Brigham and Women's Hospital, Boston, MA, USA

^4^ Harvard T.H. Chan School of Public Health, Department of Epidemiology, Boston, MA, USA

^5^ Waseda University, Faculty of Sport Sciences, Saitama, Japan

^6^ Tokyo Gas Co., Ltd., Health Promotion Center, Tokyo, Japan

**Correspondence:** [**rsloan@m.kufm.kagoshima-u.ac.jp**](mailto:rsloan@m.kufm.kagoshima-u.ac.jp)

**Supplementary Tables**

**Supplementary Table 1. Pearson correlation coefficients among each fitness and fatness variable.**

|  | Fitness-Fatness index | Cardiorespiratory fitness | Waist-to-height ratio | Waist circumference |
| --- | --- | --- | --- | --- |
| Cardiorespiratory fitness | 0.94 | ― | ― | ― |
| Waist-to-height ratio | -0.67 | -0.40 | ― | ― |
| Body mass index | -0.54 | -0.32 | 0.85 | 0.82 |

**Supplementary Table 2. Adjusted hazard ratios of incidence diabetes by potential risk factors**

| Potential risk factors | n | Man-years | Cases | Age-adjusted HR  (95%CIs) | Multivariable-adjusted HR (95%CIs) | P value |
| --- | --- | --- | --- | --- | --- | --- |
| Age ^a^  < 50  ≥ 50 | 2,673  2,341 | 16,972  9,676 | 168  183 | ―  ― | 1.00 (Referent)  1.48 (1.18 - 1.85) | ―  0.001 |
| Systolic blood pressure ^b^  <120  120-139  ≥ 140 (High BP) | 1,760  2,508  746 | 9,778  13,337  3,533 | 80  181  90 | 1.00 (Referent)  1.59 (1.22 - 2.07)  2.65 (1.96 - 3.60) | 1.00 (Referent)  1.34 (1.02 - 1.76)  2.05 (1.48 - 2.82) | ―  0.034  < 0.001 |
| Drinking habit ^c^  None  1-2 times/week  3-4 times/week  ≥5 times/week | 705  679  723  2,907 | 3,610  3,882  3,976  15,180 | 53  38  50  210 | 1.00 (Referent)  0.84 (0.55 - 1.27)  0.96 (0.65 - 1.41)  0.94 (0.69 - 1.26)  P for trend = 0.902 | 1.00 (Referent)  0.84 (0.55 - 1.28)  0.98 (0.66 - 1.45)  0.89 (0.66 - 1.21)  P for trend = 0.583 | ―  0.415  0.919  0.446 |
| Smoking habit ^d^  Nonsmokers  Past smokers  1-10 cigarettes/day  11-20 cigarettes/day  ≥21 cigarettes/day | 1,377  1,227  248  1,325  837 | 7,560  6,185  1,335  7,224  4,344 | 62  93  9  90  97 | 1.00 (Referent)  1.51 (1.09 - 2.09)  0.83 (0.41 - 1.67)  1.42 (1.03 - 1.96)  2.36 (1.71 - 3.25) | 1.00 (Referent)  1.45 (1.05 - 2.01)  0.96 (0.48 - 1.94)  1.62 (1.17 - 2.24)  2.50 (1.81 - 3.45) | ―  0.026  0.913  0.004  < 0.001 |
| Family history of diabetes ^e^  Not  Present | 4,135  879 | 22,039  4,609 | 263  88 | 1.00 (Referent)  1.63 (1.28 - 2.07) | 1.00 (Referent)  1.56 (1.23 - 1.99) | ―  < 0.001 |

^a^ Adjusted for cardiorespiratory fitness (continuous variable), waist-to-height ratio (continuous variable), body mass index (continuous variable), systolic blood pressure (continuous variable), drinking habit (none, 1-2 times/week, 3-4 times/week, and ≥5 times/week), smoking habit (nonsmokers, past smokers, 1-10 cigarettes/day, 11-20 cigarettes/day, and ≥21 cigarettes/day), and family history of diabetes (present or not).

^b^ Adjusted for age (continuous variable), cardiorespiratory fitness (continuous variable), waist-to-height ratio (continuous variable), body mass index (continuous variable), drinking habit (none, 1-2 times/week, 3-4 times/week, and ≥5 times/week), smoking habit (nonsmokers, past smokers, 1-10 cigarettes/day, 11-20 cigarettes/day, and ≥21 cigarettes/day), and family history of diabetes (present or not).

^c^ Adjusted for Adjusted for age (continuous variable), cardiorespiratory fitness (continuous variable), waist-to-height ratio (continuous variable), body mass index (continuous variable), systolic blood pressure (continuous variable), smoking habit (nonsmokers, past smokers, 1-10 cigarettes/day, 11-20 cigarettes/day, and ≥21 cigarettes/day), and family history of diabetes (present or not).

^d^ Adjusted for age (continuous variable), cardiorespiratory fitness (continuous variable), waist-to-height ratio (continuous variable), body mass index (continuous variable), systolic blood pressure (continuous variable), drinking habit (none, 1-2 times/week, 3-4 times/week, and ≥5 times/week), and family history of diabetes (present or not).

^e^ Adjusted for age (continuous variable), cardiorespiratory fitness (continuous variable), waist-to-height ratio (continuous variable), body mass index (continuous variable), systolic blood pressure (continuous variable), drinking habit (none, 1-2 times/week, 3-4 times/week, and ≥5 times/week), and smoking habit (nonsmokers, past smokers, 1-10)
